# Supplementary material for: Simple controls exceed best deep learning algorithms and reveal foundation model effectiveness for predicting genetic perturbations
Source: Bioinformatics. 2025 May 23;41(6):btaf317. doi: 10.1093/bioinformatics/btaf317 (PMC12202205; doi:10.1093/bioinformatics/btaf317)
Supplement: btaf317_Supplementary_Data [file btaf317_supplementary_data.zip › btaf317_Supplementary_Data/Final Supplement.docx]

# **Supplementary Information**

# **Simple controls exceed best deep learning algorithms and reveal foundation model effectiveness for predicting genetic perturbations**


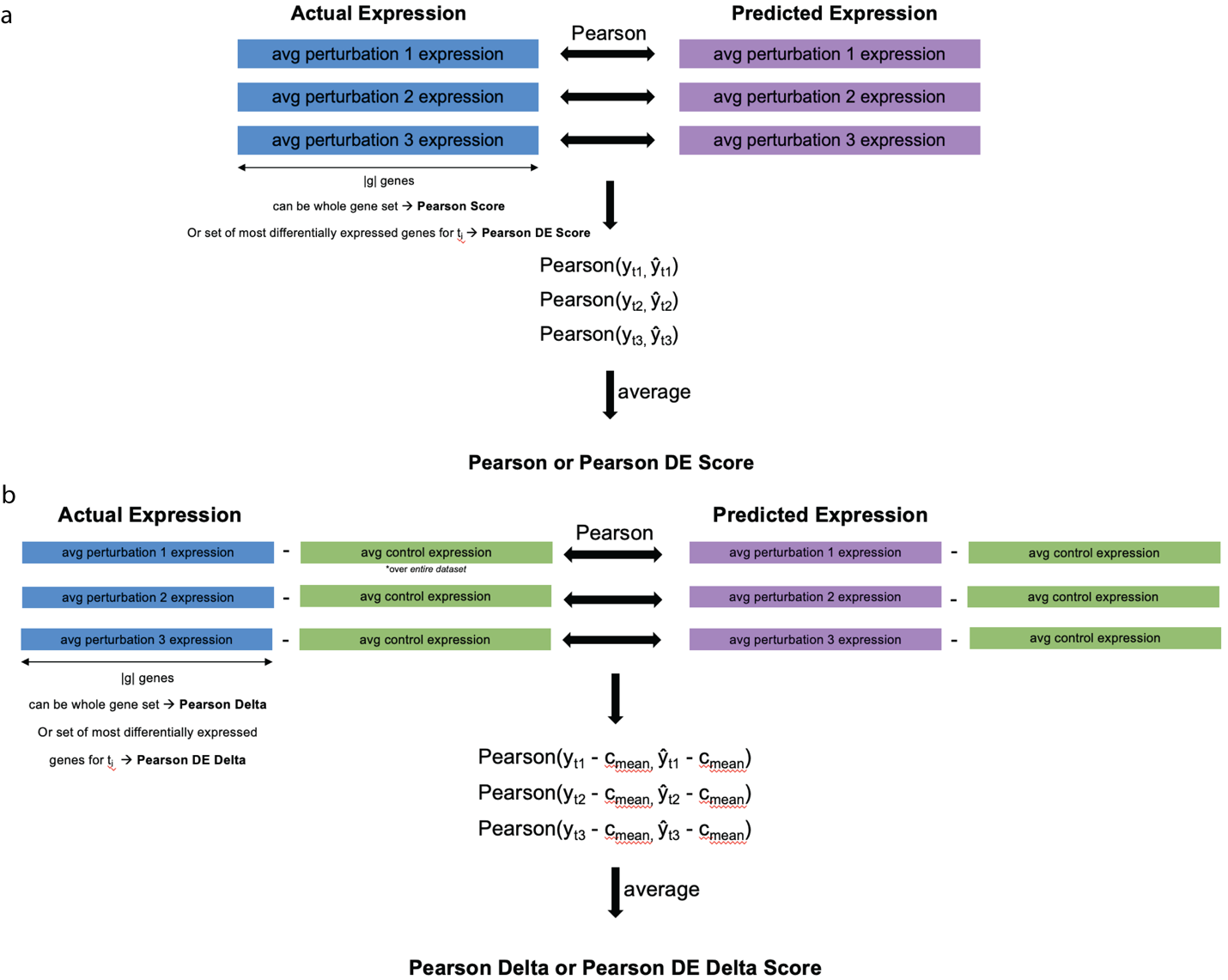


**Supplemental Figure 1: Pictorial representation of Pearson metrics for measuring post-perturbation prediction performance.**

(a) Pearson and Pearson DE scores. y_ti_ = actual expression of cells with target=t_i_.

(b) Person Delta and Pearson DE Delta scores.

**
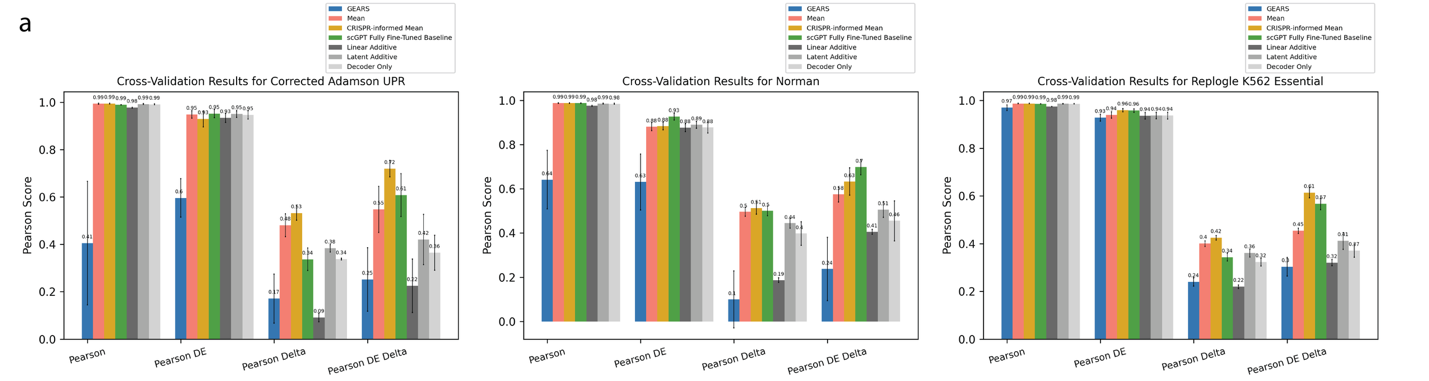
**

**Supplemental Figure 2: Four-fold cross-validation.** We split the perturbations into four random folds, and assigned two of them for training, one for validation, and one for testing. Error bars show the standard deviation of the four folds.


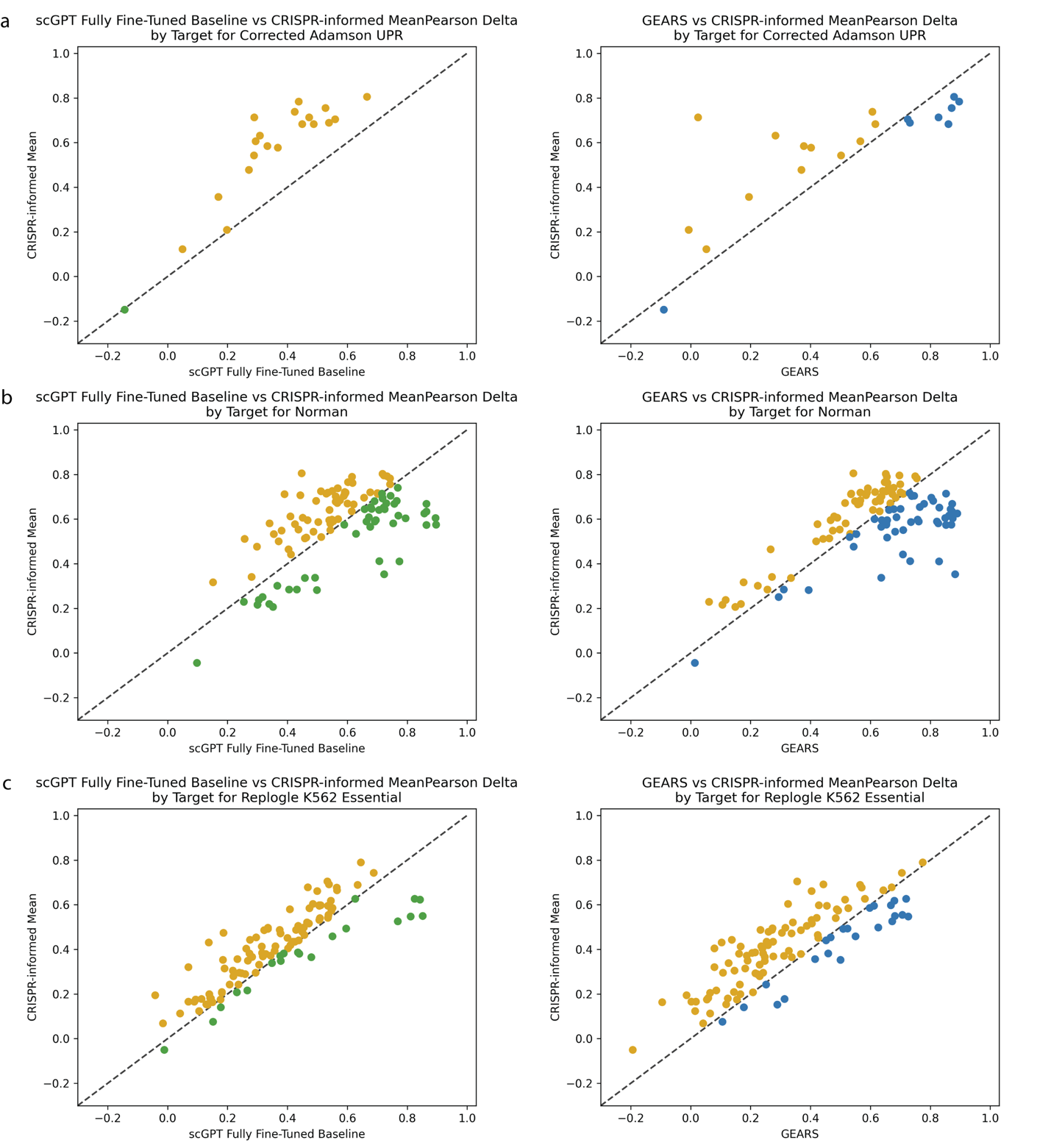


**Supplemental Figure 3: Performance by perturbation condition over the test set.** X-axis DL model (either scGPT (left) or GEARS (right), Y-axis CRISPR-informed Mean Model. Each point is a perturbation condition. We used the best performing GEARS and scGPT models of the ten independent runs to derive predictions. The color indicates which model was the most performant for that condition: CRISPR-informed model: gold, scGPT: green, GEARS: blue.

(a) Corrected Adamson UPR dataset.

(b) Norman dataset.

(c) Replogle K562 Essential dataset.


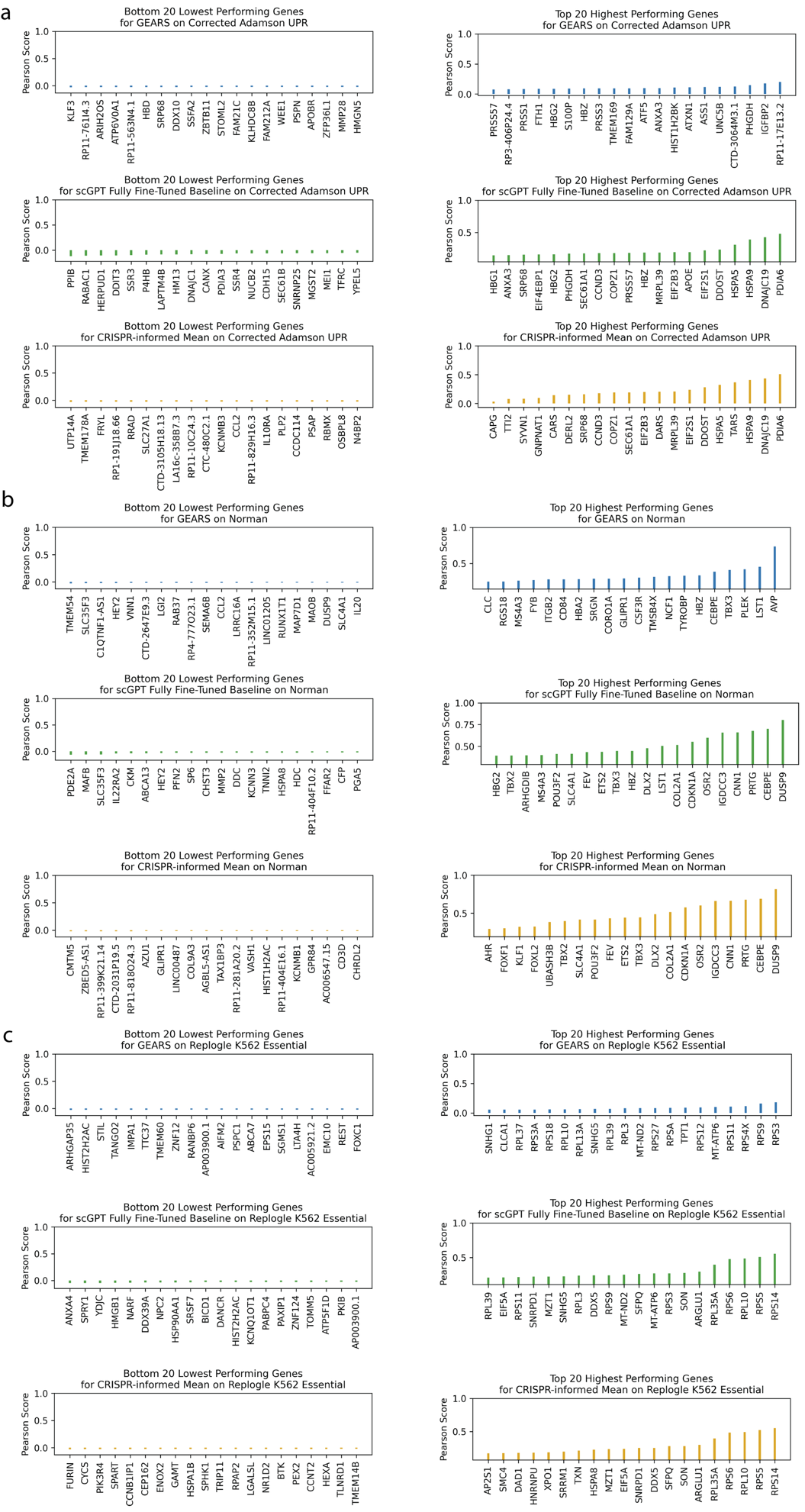


**Supplemental Figure 4: Performance by gene over test set.** Left: Bottom 20 lowest performing genes for each method. Right: top 20 highest performing. We took the Pearson correlation between the predicted and the actual expression for all conditions in the test set. We used the best performing GEARS and scGPT models of the ten independent runs to derive predictions. For the CRISPR-informed mean model, the predicted expression for gene g is the same for every cell (by definition). This resulted in a prediction vector with zero standard deviation, and hence an invalid Pearson correlation. For this plot only, we added uniform random noise in the range 0 to 1x10^-7^ to each CRISPR-informed mean prediction.

(a) Corrected Adamson UPR dataset. Gene overlap between top 20 of scGPT and CRISRP-informed mean: 12/20.

(b) Norman dataset. Gene overlap between top 20 of scGPT and CRISRP-informed mean: 15/20.

(c) Replogle K562 Essential dataset. Gene overlap between top 20 of scGPT and CRISRP-informed mean: 12/20.

**
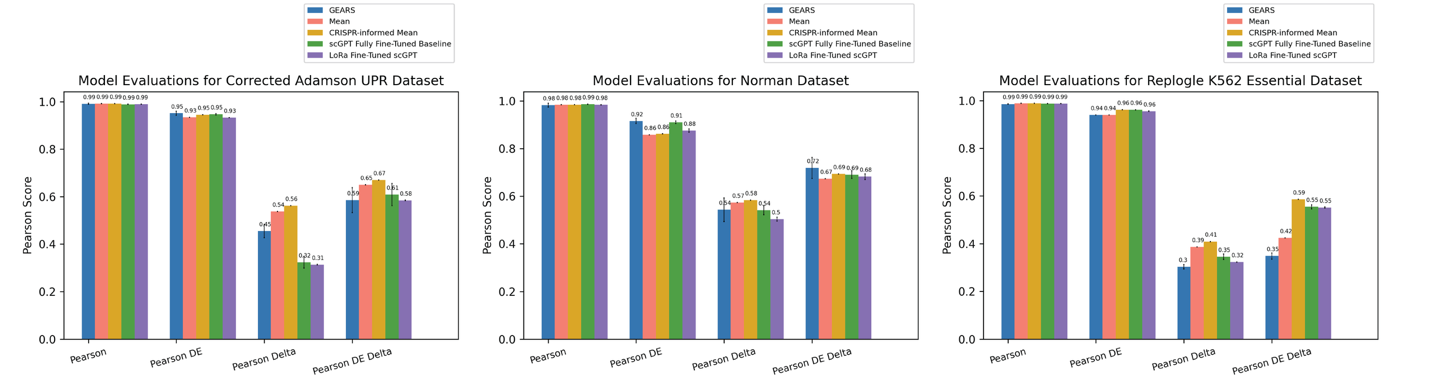
**

**Supplemental Figure 5: Performance bar charts on held-out test set comparing LoRa fine-tuned scGPT to other models.** Error bars span plus and minus one standard deviation of ten independently trained models applied to the same test set.


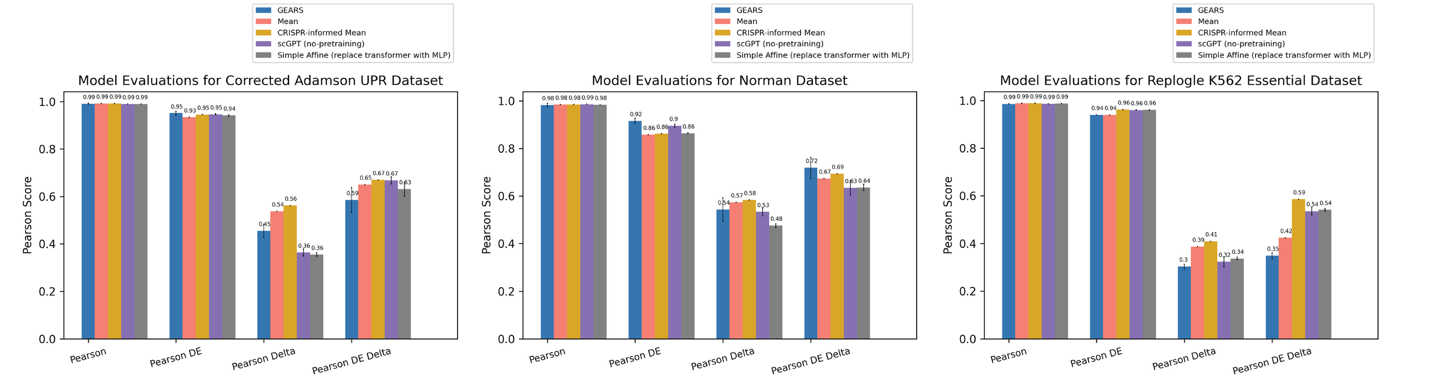


**Supplemental Figure 6: Simple Affine model with MLP replacement over test set.** We replaced the transformer block of scGPT with a simple multilayer perceptron (MLP) that had the same number of layers as the original transformer block. The MLP used a ReLU non-linear transformation, with an equivalent embedding size as the transformer block (512). Error bars show the standard deviation across ten independent model runs.


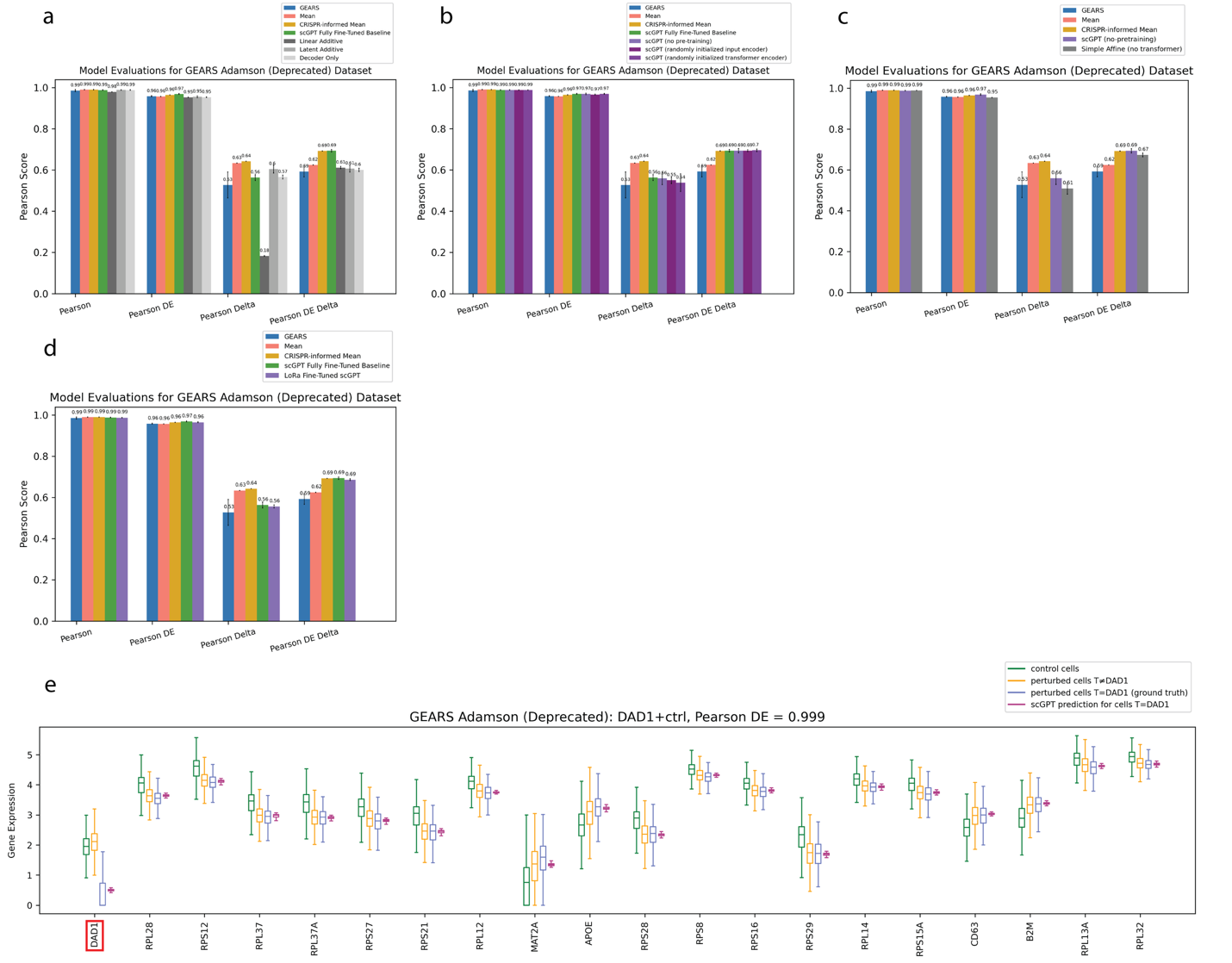


**Supplemental Figure 7: Results for the Deprecated GEARS Adamson dataset.** (a) – (d) performance bar charts on held-out test set for different models. Error bars span plus and minus one standard deviation of ten independently trained models applied to the same test set.

(a) Baseline performance across different models.

(b) Various pre-training controls.

(c) Simple affine performance.

(d) LoRa fine-tuning.

(e) Boxplots of top 20 DE genes for an example high-performing held-out perturbation condition (same perturbation as shown in the original scGPT paper^27^).


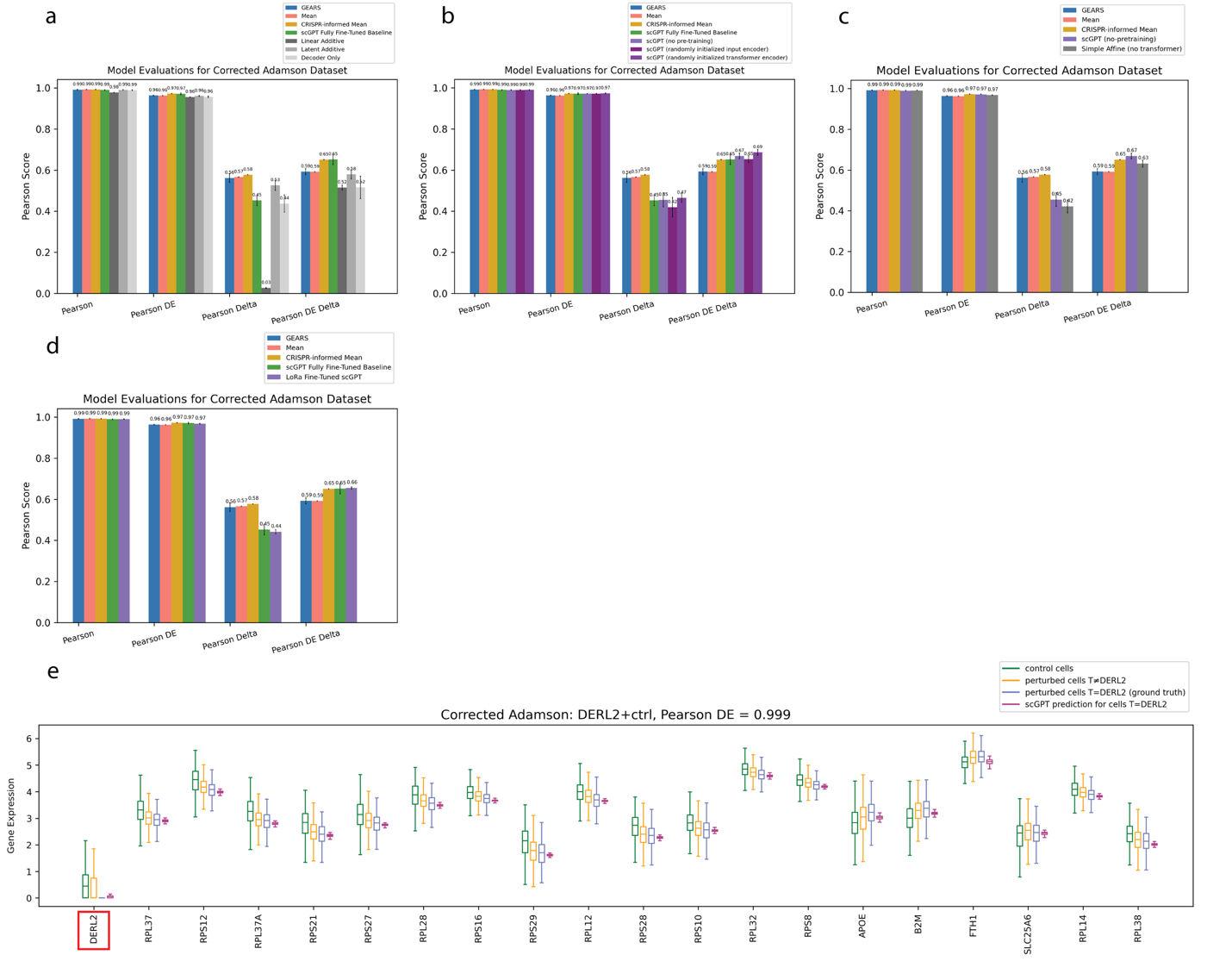


**Supplemental Figure 8: Results for the Corrected GEARS Adamson dataset.** All models were trained on the corrected combination of the three sub-experiments.

(a) – (d) performance bar charts on held-out test set for different models. Error bars span plus and minus one standard deviation of ten independently trained models applied to the same test set.

(a) Baseline performance across different models.

(b) Various pre-training controls.

(c) Simple affine performance.

(d) LoRa fine-tuning.

(e) Boxplots of top 20 DE genes for an example high-performing held-out perturbation condition.

**
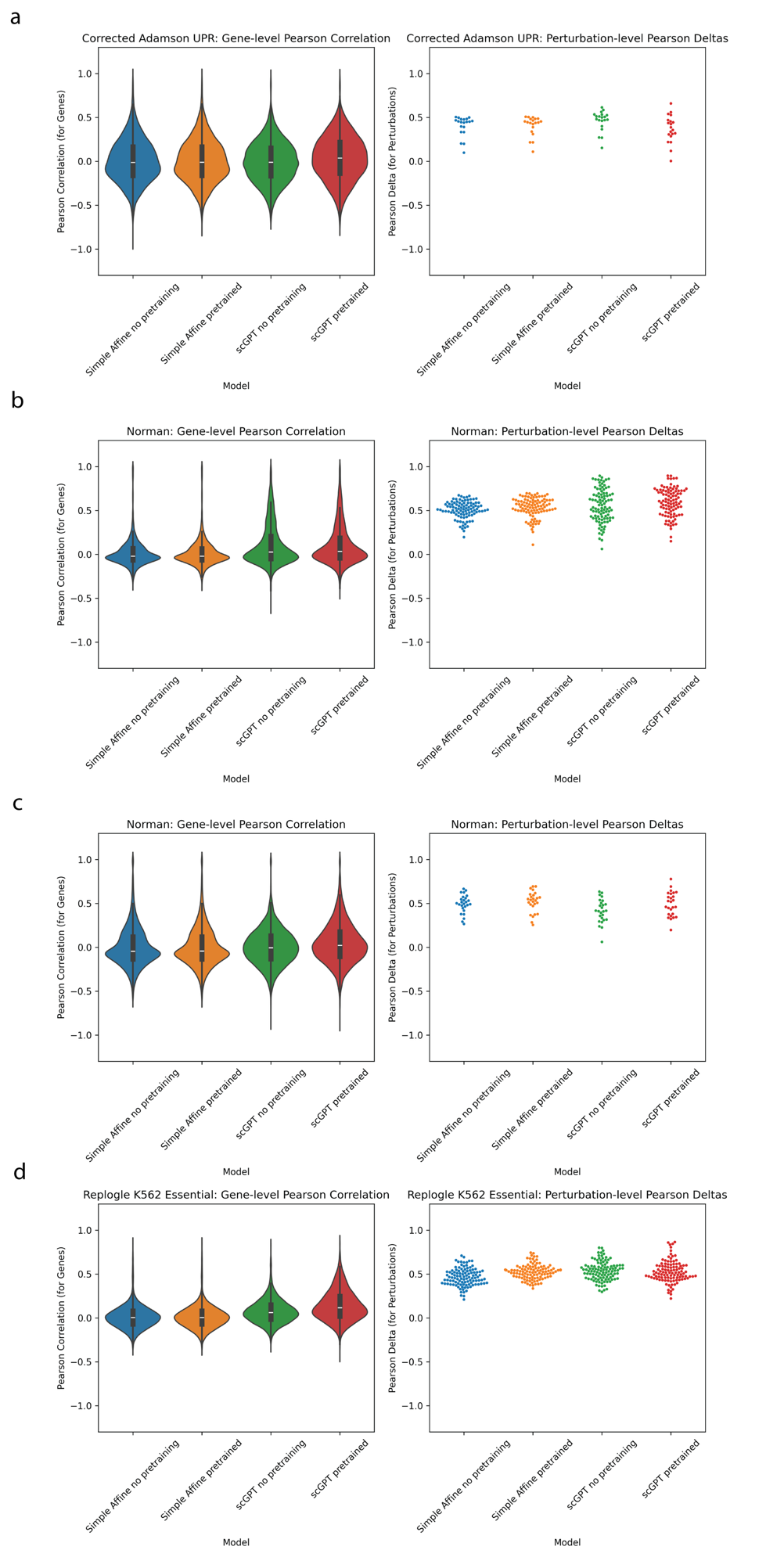
**

**Supplemental Figure 9: Comparing attention-based models to attention-free models with and without pre-training.** Plots of gene-level Pearson correlations between actual condition means and predicted means (left) and perturbation-level PD (right). Violin plots were used when data points exceeded 500 points else swarm plots. Perturbation-level PD is calculated using the actual and predicted expression of all genes for a given condition. Gene-level Pearson correlations are calculated using the transpose of those matrices, with the correlation calculated between the actual and predicted expression of each gene across all perturbations. The target gene itself is excluded because it is a highly influential point and not directly relevant to the task of downstream biological predictions. X-axis indicates which model the predictions come from: (1) Simple Affine, (2) Simple Affine with pretrained input encoder weights, (3) scGPT architecture without pretraining and (4) fully fine-tuned scGPT.

(a) Adamson UPR experiment data only

(b) Norman single and double perturbation data

(c) Norman just single perturbation data (the same gene can be present in both train and test for double perturbation data, but not single)

(d) Replogle K562 essential dataset. There is an apparent improvement in predictions using scGPT compared to the other models. The best-predicted conditions are those for which ribosomal proteins (RP) are targeted (5 conditions in the test set), and the best-predicted genes are those that change in RP targeted conditions. This observed effect may be biologically relevant learning resulting from the combination of the scGPT architecture and the pre-training, but we cannot rule out the possibility that it could be the result of an unknown or unobserved technical artifact.

**
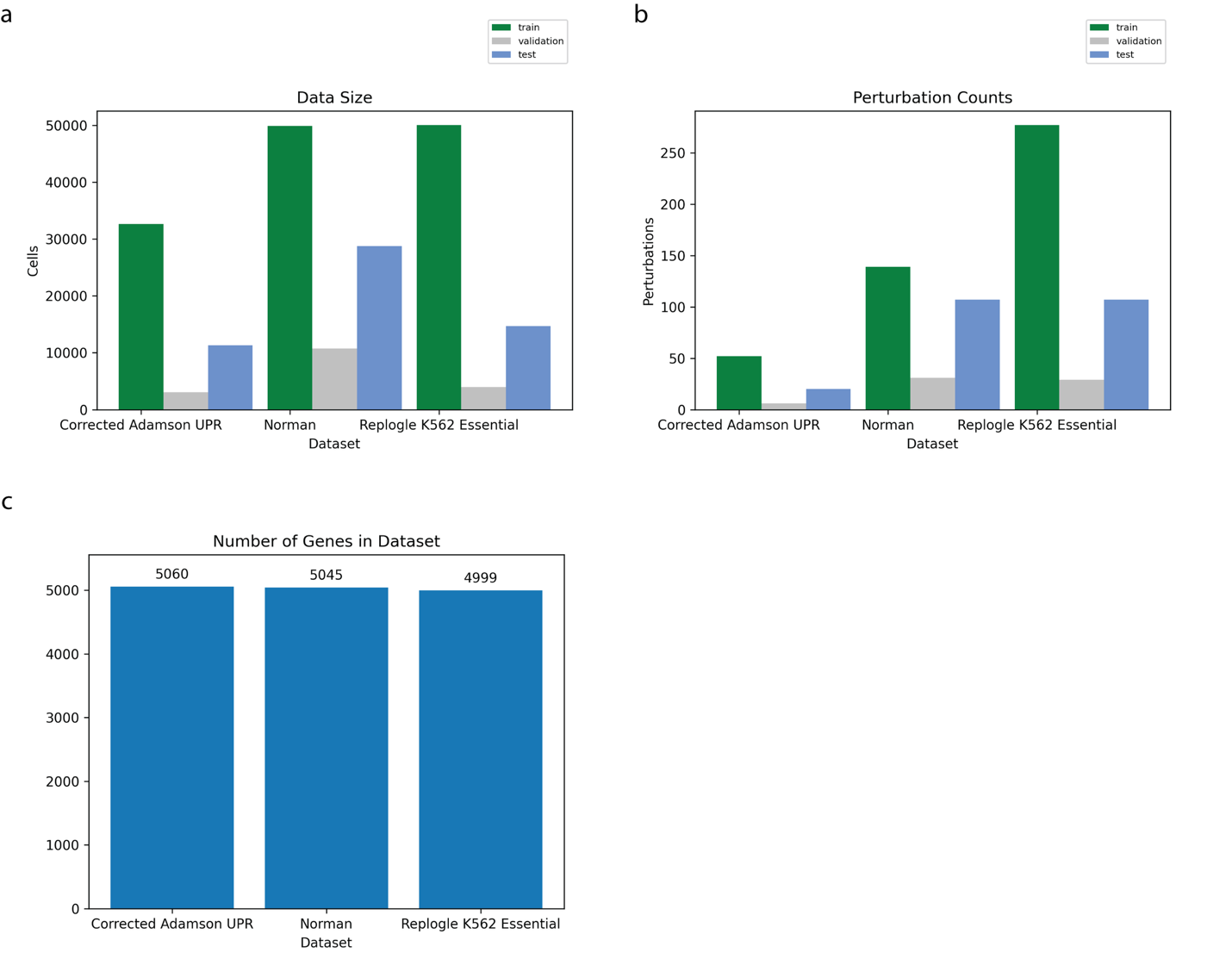
**

**Supplemental Figure 10: Cell, perturbation, and gene counts.**

(a) Cell counts for each dataset for each data split.

(b) Unique perturbation counts. Combination perturbations in the Norman dataset are counted as unique.

(c) Number of genes in each dataset.
