# Supplementary figures and images for: Simple controls exceed best deep learning algorithms and reveal foundation model effectiveness for predicting genetic perturbations
Source: Bioinformatics. 2025 May 23;41(6):btaf317. doi: 10.1093/bioinformatics/btaf317 (PMC12202205; doi:10.1093/bioinformatics/btaf317)

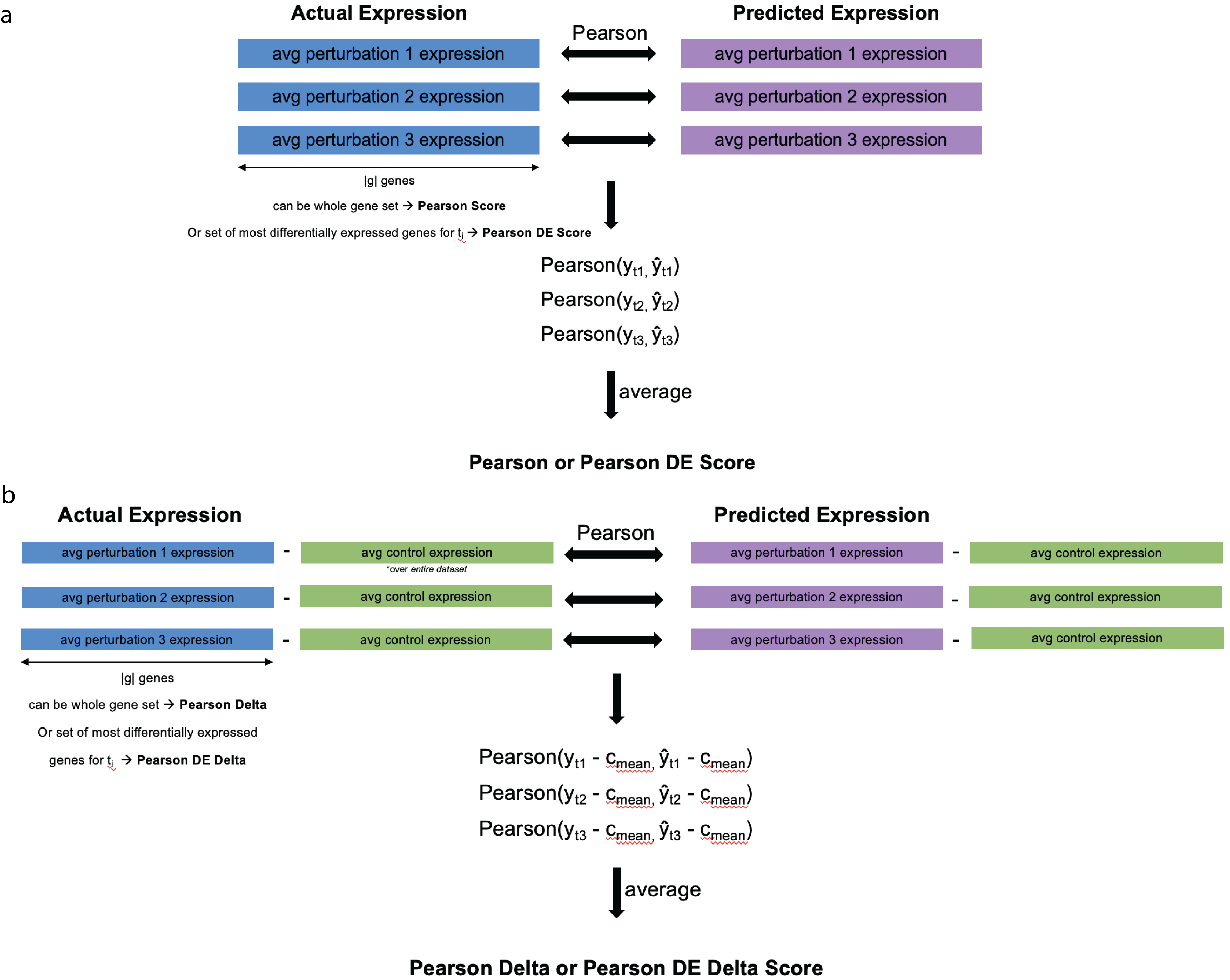

Supplement: btaf317_Supplementary_Data [file btaf317_supplementary_data.zip › btaf317_Supplementary_Data/FigureS1.png]

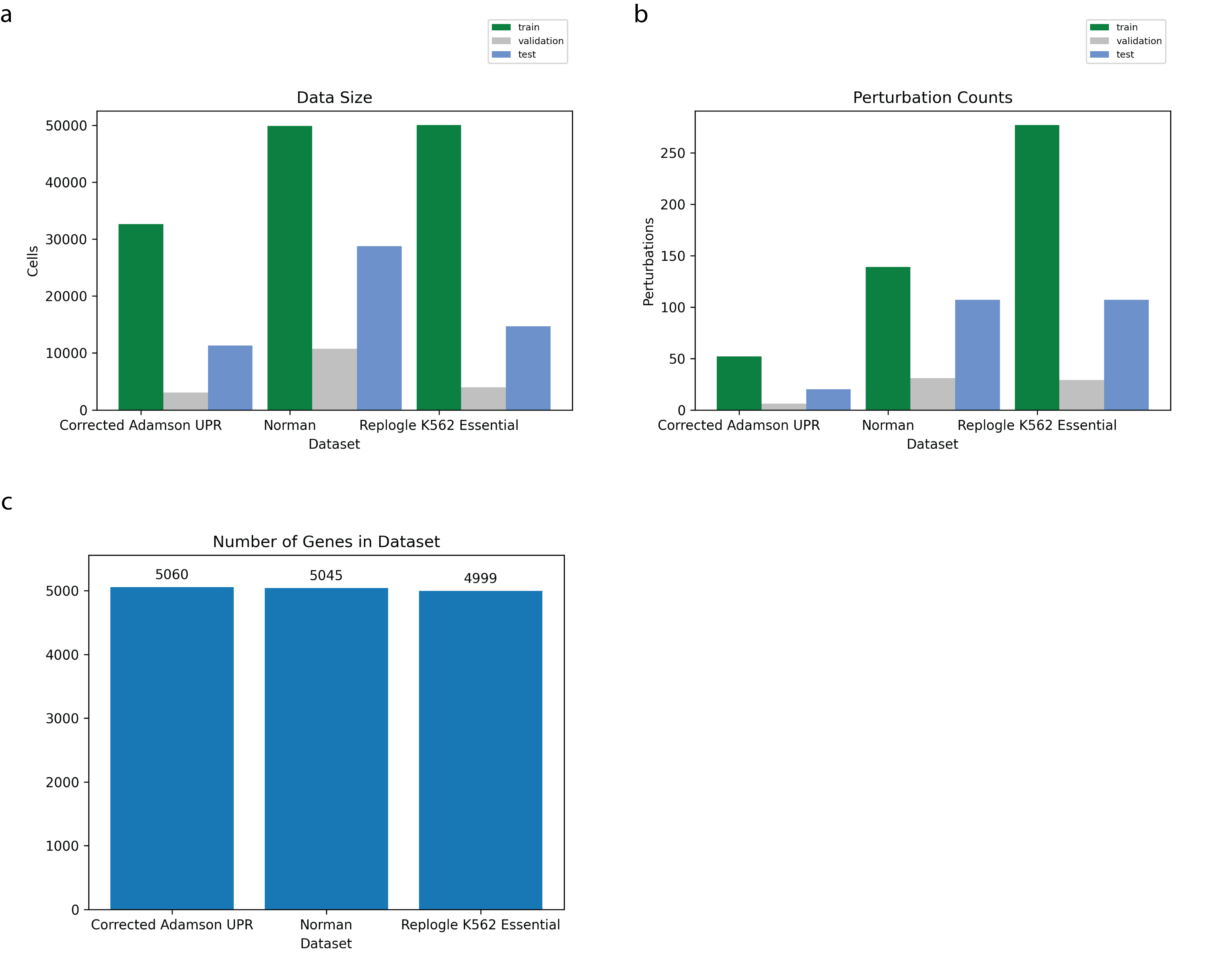

Supplement: btaf317_Supplementary_Data [file btaf317_supplementary_data.zip › btaf317_Supplementary_Data/FigureS10.png]

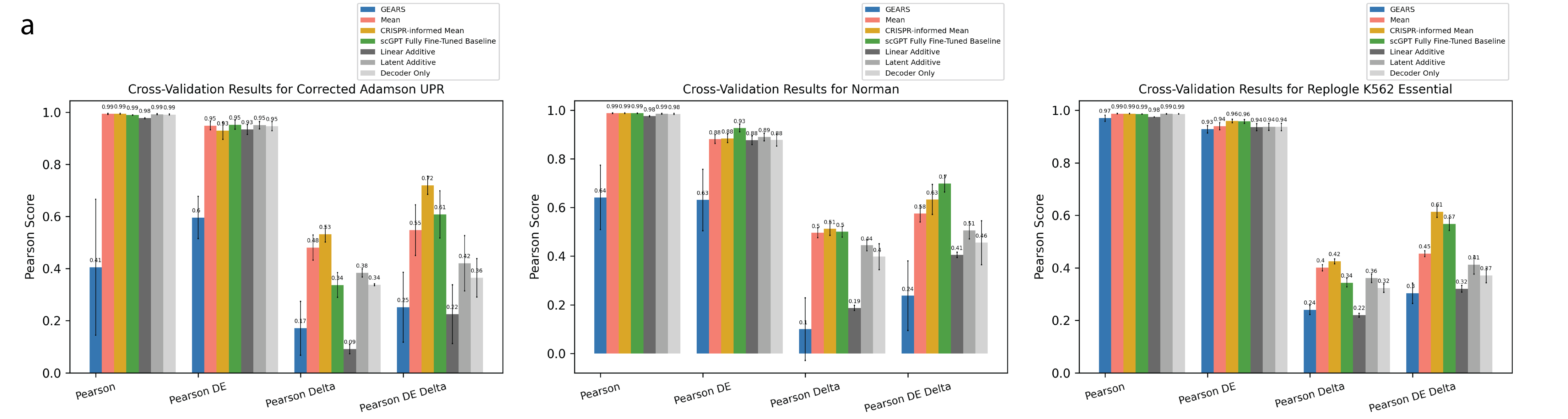

Supplement: btaf317_Supplementary_Data [file btaf317_supplementary_data.zip › btaf317_Supplementary_Data/FigureS2.png]

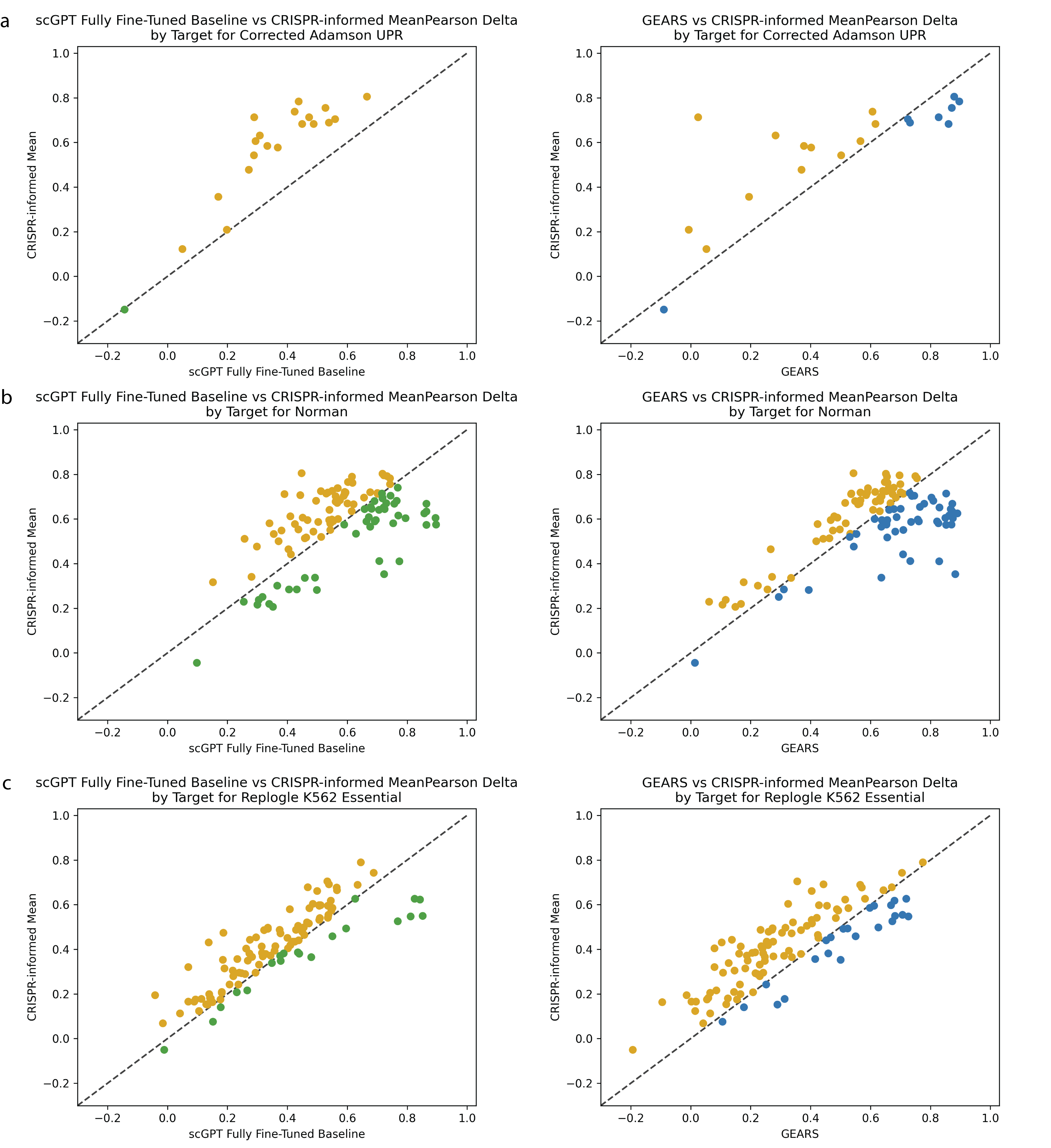

Supplement: btaf317_Supplementary_Data [file btaf317_supplementary_data.zip › btaf317_Supplementary_Data/FigureS3.png]

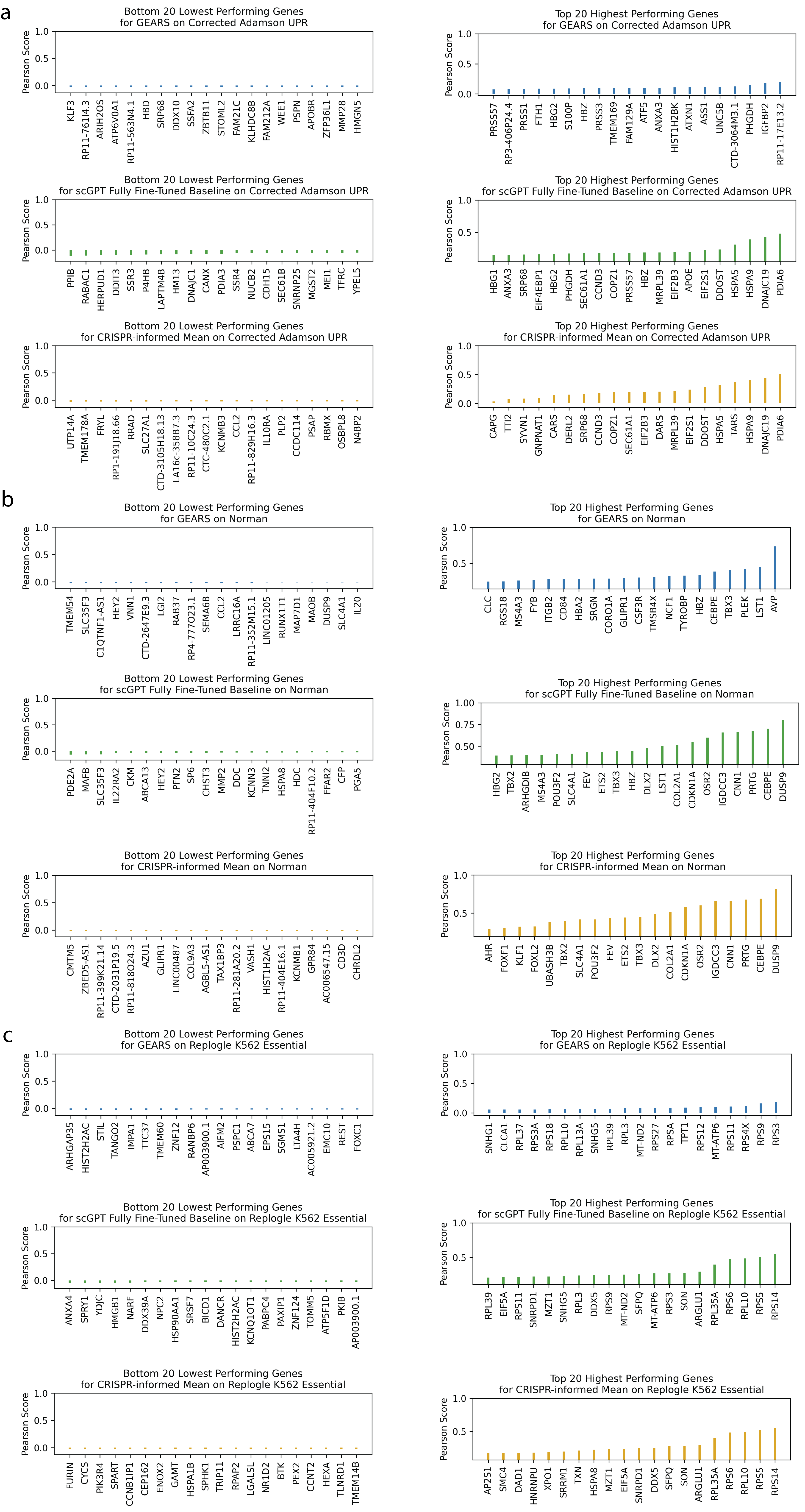

Supplement: btaf317_Supplementary_Data [file btaf317_supplementary_data.zip › btaf317_Supplementary_Data/FigureS4.png]

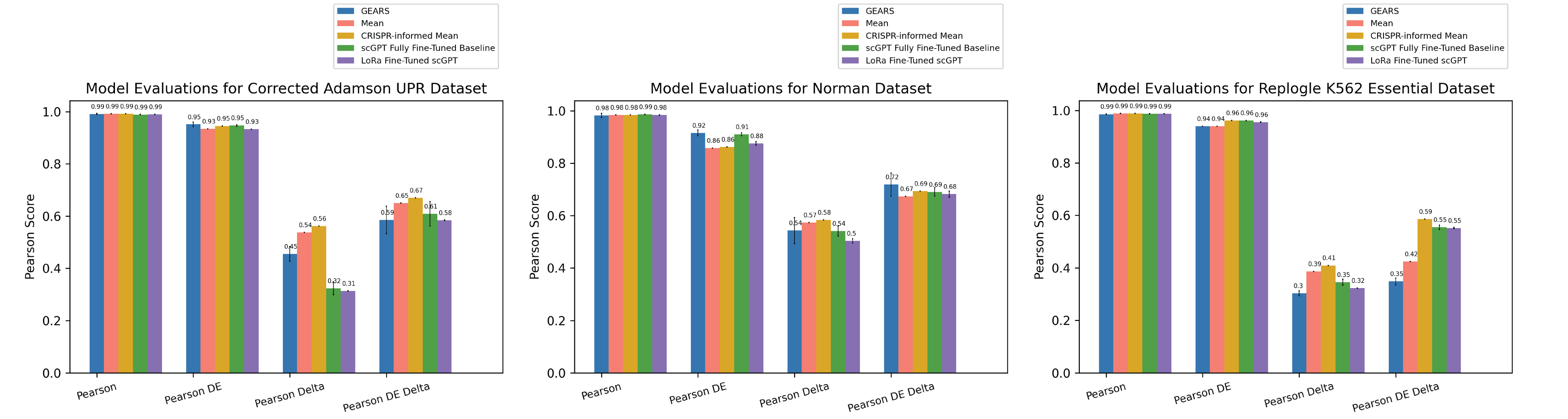

Supplement: btaf317_Supplementary_Data [file btaf317_supplementary_data.zip › btaf317_Supplementary_Data/FigureS5.png]

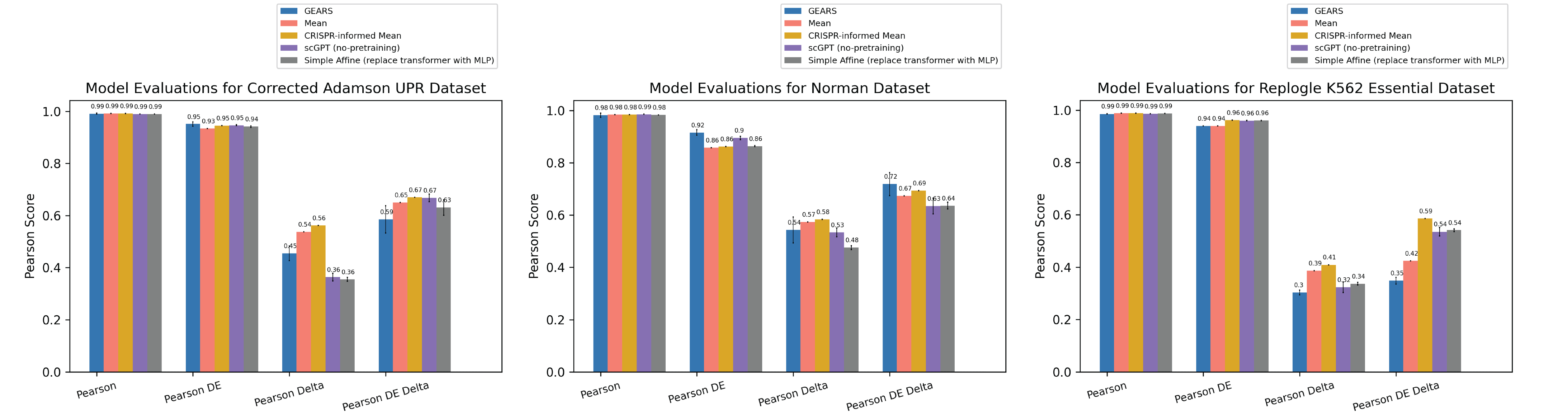

Supplement: btaf317_Supplementary_Data [file btaf317_supplementary_data.zip › btaf317_Supplementary_Data/FigureS6.png]

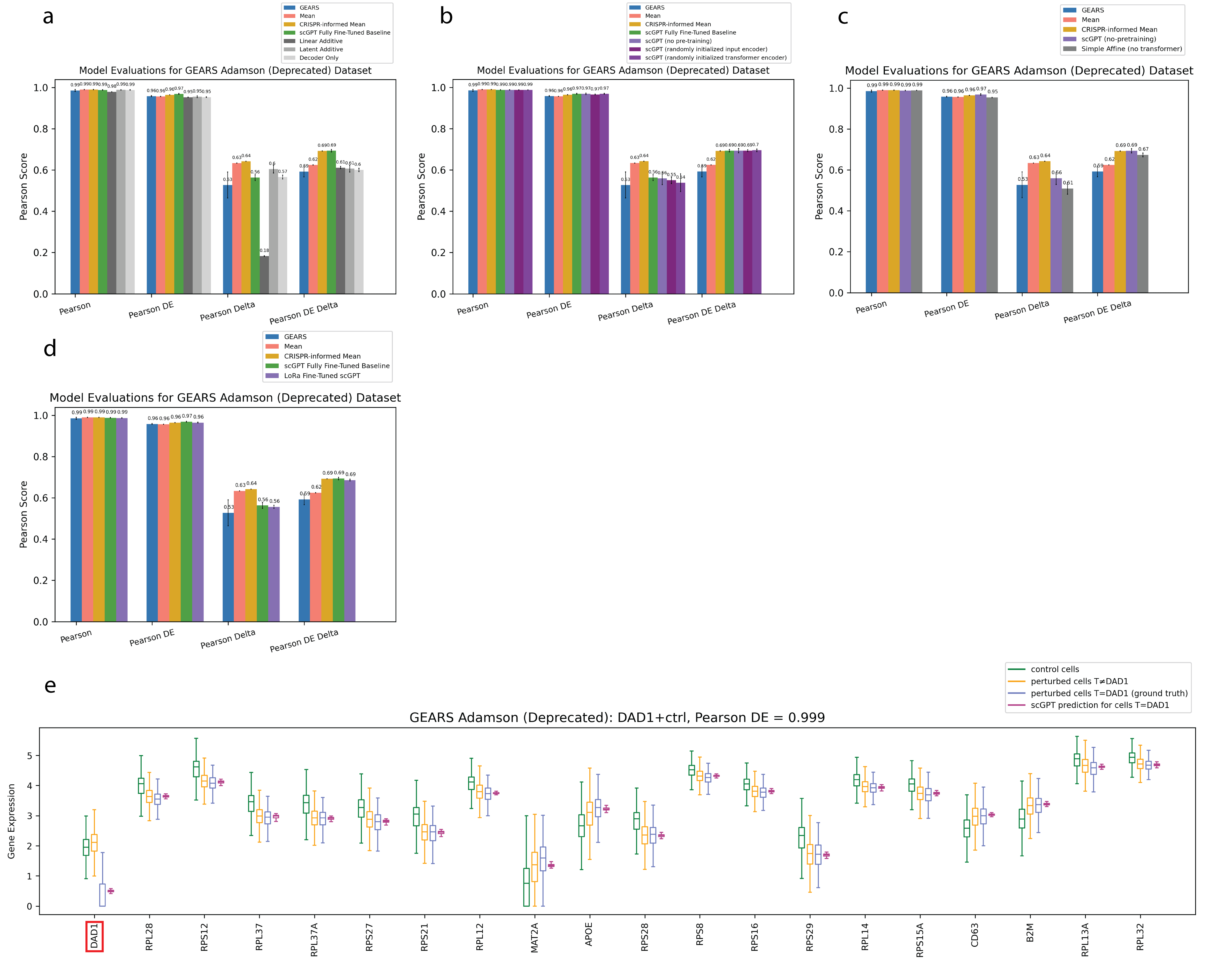

Supplement: btaf317_Supplementary_Data [file btaf317_supplementary_data.zip › btaf317_Supplementary_Data/FigureS7.png]

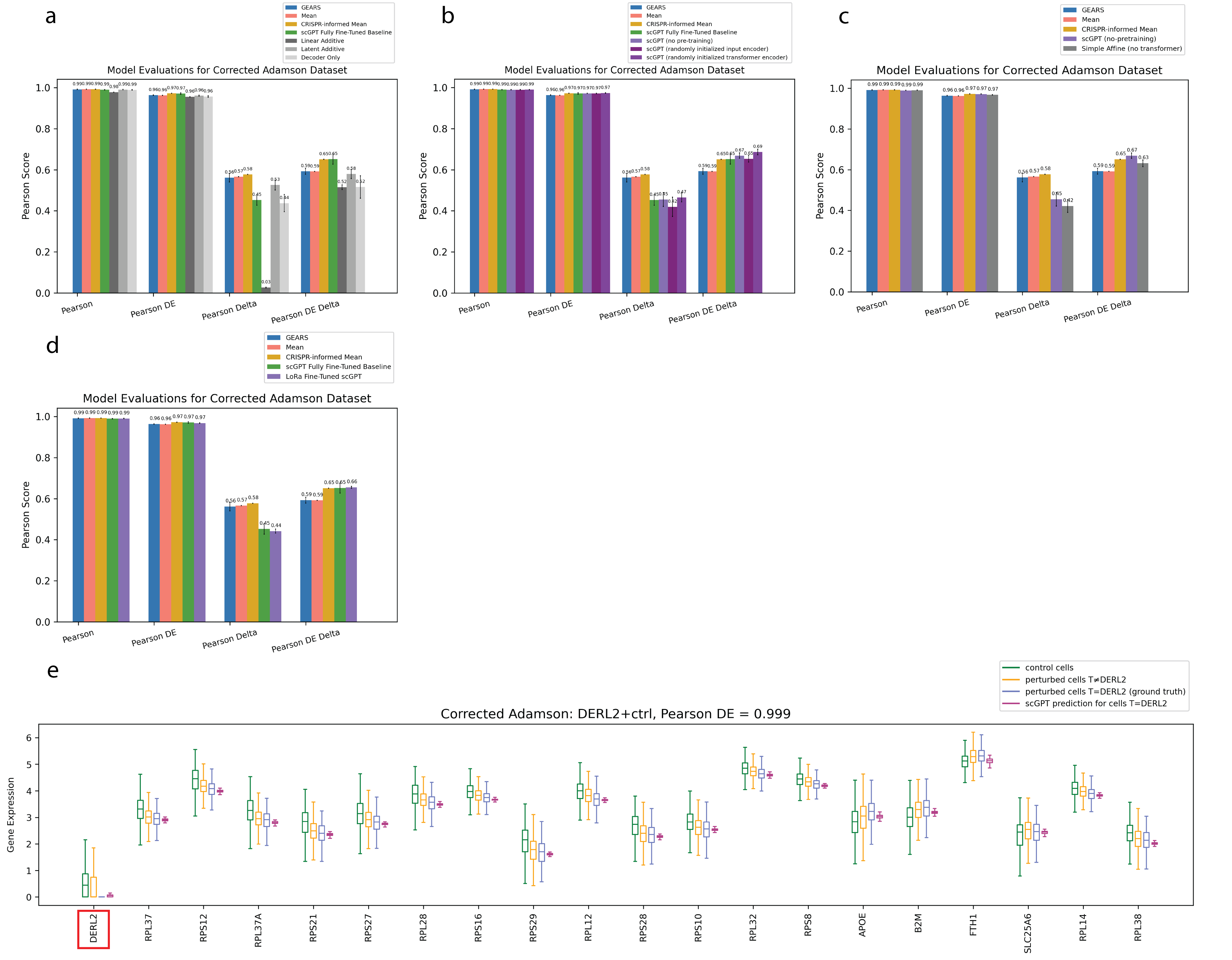

Supplement: btaf317_Supplementary_Data [file btaf317_supplementary_data.zip › btaf317_Supplementary_Data/FigureS8.png]

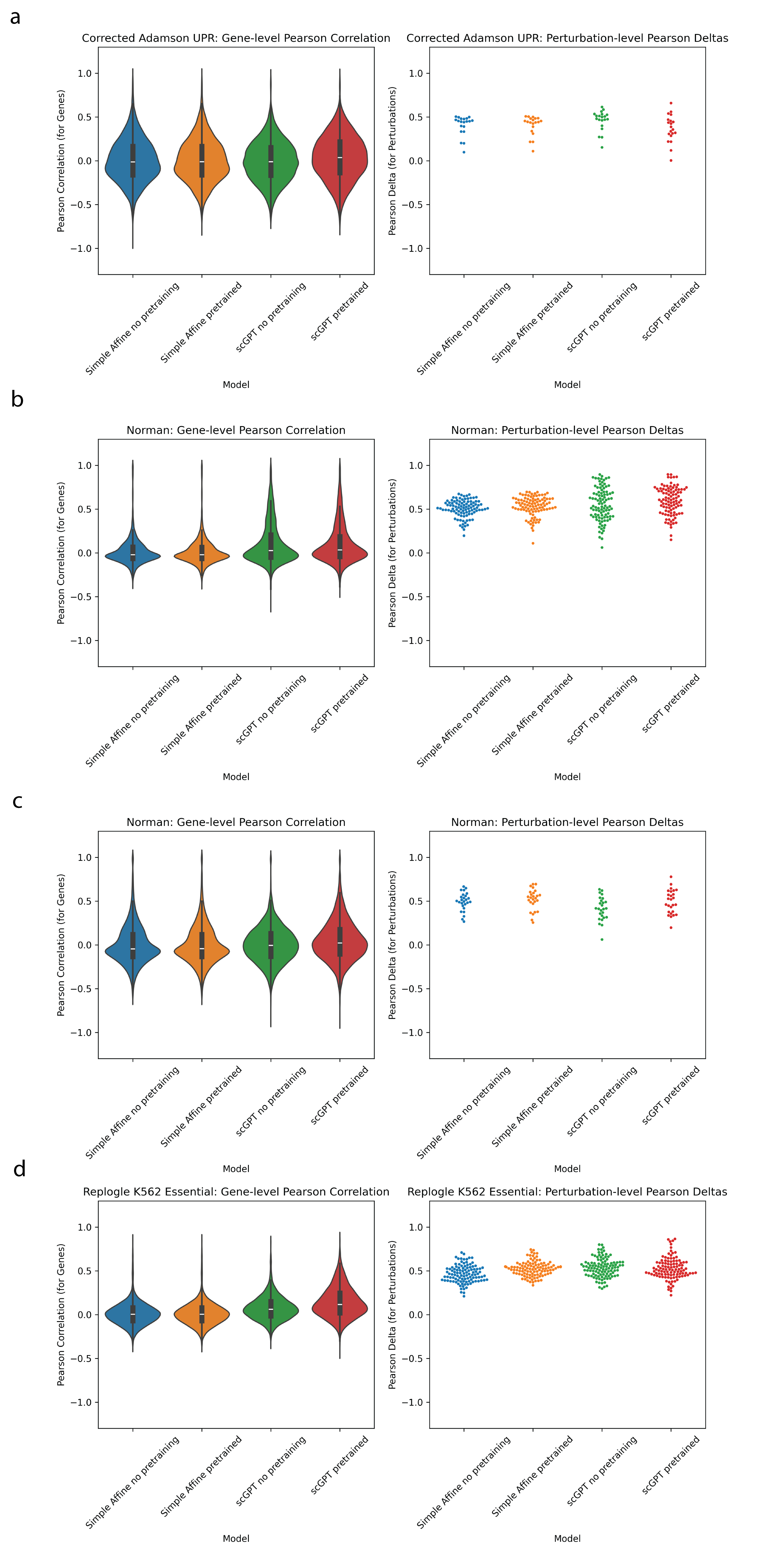

Supplement: btaf317_Supplementary_Data [file btaf317_supplementary_data.zip › btaf317_Supplementary_Data/FigureS9.png]
